# Dental Department, So. Med. College

**Published:** 1889-09

**Authors:** 


SEE DENTAL DEPARTMENT of Southern Medical College on insert in reading matter. This school is now thoroughly oaganized and equipped in all departments. The chairs are ably filled, and the advantages given the student are of a superior order.

